# Severe infections in patients with lymphoproliferative diseases treated with new targeted drugs: A multicentric real‐world study

**DOI:** 10.1002/cam4.4293

**Published:** 2021-09-23

**Authors:** Maria Stefania Infante, Ana Fernández‐Cruz, Lucia Núñez, Cecilia Carpio, Ana Jiménez‐Ubieto, Javier López‐Jiménez, Lourdes Vásquez, Raquel Del Campo, Samuel Romero, Carmen Alonso, Daniel Morillo, Margarita Prat, José Luis Plana, Paola Villafuerte, Gabriela Bastidas, Ana Bocanegra, Ángel Serna, Rodrigo De Nicolás, Juan Marquet, Carmen Mas‐Ochoa, Raúl Cordoba, Julio García‐Suárez, Alessandra Comai, Xavier Martín, Mariana Bastos‐Oreiro, Cristina Seri, Belén Navarro‐Matilla, Armando López‐Guillermo, Joaquín Martínez‐López, José Ángel Hernández‐Rivas, Isabel Ruiz‐Camps, Carlos Grande, Grupo Español de Linfomas y Trasplante Autólogo de Medula Ósea (GELTAMO)

**Affiliations:** ^1^ Hematology Department Hospital Universitario Infanta Leonor Madrid Spain; ^2^ Infectious Diseases Department Hospital Universitario Puerta de Hierro‐Majadahonda Spain; ^3^ Hematology Department Hospital Universitario Puerta de Hierro‐Majadahonda Madrid Spain; ^4^ Hematology Department Hospital Vall de Hebrón Barcelona Spain; ^5^ Hematology Department, Hospital 12 de Octubre Complutense University CNIO Madrid Spain; ^6^ Hematology Department Hospital Universitario Ramón y Cajal Madrid Spain; ^7^ Hematology Department Hospital Clínico Universitário de Salamanca (CAUSA/IBSAL) Salamanca Spain; ^8^ Hematology Department Hospital Son Llàtzer Palma Spain; ^9^ Hematology Department Hospital Universitario y Politécnico La Fe Valencia Spain; ^10^ Hematology Department Hospital Arnau de Vilanova Valencia Spain; ^11^ Hematology Department Fundación Jimenez Diaz University Hospital Health Research Institute IIS‐FJD Madrid Spain; ^12^ Hematology Department Hospital Sant Pau y Santa Tecla Tarragona Spain; ^13^ Hematology Department Hospital del Vendrell Vendrell Spain; ^14^ Hematology Department Hospital Universitário Príncipe de Astúrias Alcalá de Henares Spain; ^15^ Hematology Department Institut d’Investigacions Biomèdiques August Pi i Sunyer (IDIBAPS) Barcelona Spain; ^16^ Hematology Department Hospital General de Catalunya Spain; ^17^ Hematology Department Hospital de Cruces Barakaldo Spain; ^18^ Hematology Department Hospital General Gregorio Marañon Madrid Spain; ^19^ Hematology Department Hospital Central de la Defensa Gómez Ulla Madrid Spain; ^20^ Hematology Department Hospital Clínic of Barcelona Barcelona Spain; ^21^ Infectious Diseases Department Hospital Vall de Hebrón Barcelona Spain; ^22^ Hematology Department Clínica Universidad de Navarra Madrid Spain

**Keywords:** infectious diseases, infectious risk, lymphoproliferative disease, prophylaxis, targeted drugs

## Abstract

**Background:**

Lymphoid neoplasms treatment has recently been renewed to increase antitumor efficacy and conventional chemotherapies toxicities. Limited data have been published about the infection risk associated with these new drugs, therefore this study analyzes the infectious complications in patients with lymphoproliferative diseases (LPD) treated with monoclonal antibodies (obinutuzumab, ofatumumab, brentuximab, nivolumab, or pembrolizumab), BTK inhibitors (ibrutinib and acalabrutinib), PI3K inhibitors (idelalisib) and BCL2 inhibitors (venetoclax).

**Methods:**

Multicenter retrospective study of 458 LPD patients treated with targeted therapies in real‐life setting, in 18 Spanish institutions, from the time of their commercial availability to August 2020.

**Results:**

Severe infections incidence was 23% during 17‐month median follow‐up; cumulative incidence was higher in the first 3–6 months of targeted drug treatment and then decreased. The most frequent etiology was bacterial (54%). Nine (6%) Invasive fungal infections (IFI) were observed, in its majority in chronic lymphocytic leukemia (CLL) patients treated predominantly with ibrutinib. Significant risk factors for severe infection were: severe lymphopenia (*p* = 0.009, OR 4.7, range 1.3–1.7), combined targeted treatment vs single agent treatment (*p* = 0.014 OR 2.2 range 1.1–4.2) and previous rituximab (*p* = 0.03 OR 1.8, range 1.05–3.3). Infection‐related mortality was 6%. In 22% of patients with severe infections, definitive discontinuation of the targeted drug was observed.

**Conclusion:**

A high proportion of patients presented severe infections during follow‐up, with non‐negligible attributable mortality, but infection incidence is not superior to the one observed during the chemotherapy era. In selected cases with specific risk factors for infection, antimicrobial prophylaxis should be considered.

## Graphical abstract

Our data describe the infection risk associated with the use of targeted drugs in the treatment of lymphoproliferative diseases in real‐life setting and suggest some infection risk factors that could identify the need for antimicrobial prophylaxis in a selected group of patients.

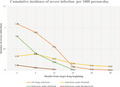

## 1 INTRODUCTION

In recent years, there has been a renewal of therapeutic tools in the treatment of lymphoid neoplasms with the appearance of different targeted drugs, among others, against B‐cell receptor or lymphocytic expression antigens signalization way. The increasing use of targeted therapies^1^ aims to increase antitumor efficacy beyond that of conventional chemotherapies, in addition to avoiding the latter's toxicity, which adds to the intrinsic immunological dysfunction of the disease itself.^2^, ^3^

Ibrutinib's main target is Bruton's tyrosine kinase (BTK), a non‐receptor tyrosine kinase from the Tec family that has a critical role in B‐cell proliferation. Ibrutinib not only inhibits BTK potently and irreversibly, but it also inhibits other related Tec family kinases, and off‐target inhibition appears to contribute to specific ibrutinib toxicities.^4^, ^5^ It also regulates other cells of the immune system, such as receptor‐mediated phagocytosis of *Candida albicans* ^6^, ^7^ in macrophages. A “net state of immunosuppression” has been proposed, which includes factors affecting the adaptive and innate immune system, disease‐related factors, aging, comorbidities, immunosuppressive drugs, and possibly a genetic predisposition as contributing to ibrutinib‐associated IFI.^8^ Cases of invasive fungal infections (IFI) including brain aspergillosis,^9^, ^10^ *Pneumocystis jirovecii* pneumonia (PJP), or cryptococcosis^11^, ^12^ have been described in patients during the first months of ibrutinib treatment^3^, ^8^: the latest findings describe an immunomodulatory effect of ibrutinib that rapidly impair innate immune cell functions, while concomitantly restoring an effective and protective adaptive immune response to fungal infection.^13^

In addition, other opportunistic infections such as miliary tuberculosis^14^ (TBC), disseminated herpes zoster^15^ (HZV), and hepatitis B reactivation^16^ (HBV) have been reported.

Acalabrutinib is a second‐generation BTK inhibitor that, unlike ibrutinib, has no activity on ITK (Interleukin‐2‐Inductible‐T‐cell kinase) or other kinases. Consequently, its administration could potentially reduce the risk of side effects and toxicities^17^: a phase 2 study of 124 patients with refractory or relapsed (R/R) mantle cell lymphoma (MCL) describes 55% of infections, with 5% pneumonias, 1 PJP, and 1 cytomegalovirus CMV reactivation.^18^ Idelalisib is an oral inhibitor of PI3K. Inhibition of PI3K could alter the function of regulatory CD4 + T lymphocytes, which, in addition to accounting for the immune‐mediated toxicities of the drug (colitis and hepatitis),^19^ appears to be involved in the response to infections. In a study of relapsed CLL, idelalisib showed 7.4% incidence of death due to fungal infection like PJP or CMV reactivation^20^ (vs 3.5% of the alternative branch).

Venetoclax is a BCL2 inhibitor that, despite an incidence of severe infections of 17% and grade 4 neutropenia in 41% of the patients in a stage I trial in R/R CLL,^21^ does not show a higher incidence of IFI or CMV reactivation.^22^

Among monoclonal antibodies used in the treatment of lymphoid malignancies, nivolumab and pembrolizumab are PD1‐inhibitors. In melanoma studies, infection risk is associated with corticosteroids and anti‐TNF treatment used to manage their immune‐mediated toxicities (pneumonitis, colitis, hepatitis etc.); there is still little published data on their use in lymphoid neoplasms.^23^, ^24^ Conjugated monoclonal antibody brentuximab vedotin is associated with an increased risk of neutropenia and VZV and HSV infections (1%–10% incidence) are described as a common effect.^25^ Cases of progressive multifocal leukoencephalopathy (PML) have been described in patients treated with brentuximab.^26^ The Gallium trial has shown that the substitution of rituximab by obinutuzumab anti‐CD20 type II antibody in association with immunochemotherapy in the treatment of first line FL improves progression‐free survival. The incidence of infections in the obinutuzumab branch was slightly higher than in the rituximab branch (77.3% vs. 70%).^27^

Treatment with the new molecules is becoming increasingly common in routine clinical practice, but infection incidence while receiving targeted therapies is extrapolated generally from clinical trials^28^ and real‐world data are lacking. Most existing studies are focused on risk of opportunistic infections in patients treated with ibrutinib.^29^ There are no clear recommendations regarding anti‐infective prophylaxis in these patients.

The aim of the present study is, in a real‐world setting, to describe the infectious complications in patients with indolent or aggressive LPD treated with targeted drugs in routine clinical practice, to identify additional factors of infectious risk in these patients and to propose, according to the infectious risk, which patients would benefit from close monitoring and targeted anti‐infective prophylaxis.

## 2 METHODS

The electronic medical records of all patients ≥18 years of age diagnosed with lymphoid cancer (including CLL, NHL, and HL), who were treated with new drugs, either as monotherapy or in combination with other drugs, were reviewed since their use was available in clinical practice from March 2011 to August 2020, in 19 Spanish academic and general hospitals.

Patient demographics, type of underlying cancer, new drug exposure and treatment duration, prior and concurrent cancer treatments, as well as clinical outcomes were collected. The following potential risk factors for infection were recorded: diabetes, liver disease, previous cancer, previous HSCT, heavily pretreated hemopathy (>3 prior lines of treatment), previous exposure to fludarabine, rituximab, bendamustine or alemtuzumab, aggressive vs. indolent LPD, combined vs single treatment, use of adjunctive corticosteroids, receipt of antimicrobial prophylaxis, presence of neutropenia or lymphopenia at any time during therapy.

Receipt of corticosteroids was defined as receipt of an average daily dose equivalent to ≥20 mg of prednisone at any time from initiation of new drug treatment to its discontinuation. Neutropenia was defined as an absolute neutrophil count ≤1.5 × 10^9^/L (<500/μl severe) and lymphopenia as an absolute lymphocyte count ≤1.8 × 10^9^/L (<800/μl severe) at any time during the course of therapy.

Infections were identified by reviewing patient medical record, laboratory data, imaging studies and histopathological or cytology results when available. For cases with microbiological and/or radiological findings suggestive of infection, we further reviewed the clinical chart to confirm the presence of associated symptoms to exclude mere colonization and ascertain clinical outcomes. The source of infection was classified according to clinical and microbiological criteria following the Centers for Disease Control (CDC) guidelines.^30^ Severe infection was defined as an infection that required hospitalization and/or parenteral antimicrobial therapy that occurred at any time from initiation of the new drug until 30 days after its discontinuation. IFIs were defined as per the revised 2008 European Organization for Research and Treatment of Cancer/Invasive Fungal Infections Cooperative Group guidelines.^27^ The cause of death was determined by consensus agreement among the investigators.

### 2.1 DATA ANALYSIS

Data were processed using REDCap (Research Electronic Data Capture) tools hosted at GELTAMO offices. Cases with and without severe infection were compared regarding baseline characteristics and targeted treatment exposure. Quantitative variables were expressed as means (standard deviation) or as medians with interquartile range (IQR), as appropriate; qualitative variables were expressed as frequency and percentage. Continuous variables were compared using the *t*‐test, and categorical variables were compared using the χ^2^ test or Fisher exact test when the χ^2^ test was not appropriate. In addition, infection incidence per 1000 person‐year was computed to incorporate the multiple severe infections per patient. Adjusted odds ratios (ORs) were calculated using logistic regression analysis to compare cases and controls. Multivariable stepwise logistic regression analysis was performed including variables with a *p*‐value <0.05 in the univariate analysis. All statistical analyses were performed using IBM SPSS Statistics for Windows, version 22 (IBM Corp.). The study and the case report form were approved by the local institutional review board and ethics committee (Institutional Review Board of University Hospital Gregorio Marañón, Madrid, Spain, GEL‐IBR‐2018–02).

## 3 RESULTS

A total of 462 patients with LPD received targeted drugs during the study period: 4 patients were excluded for missing data during the follow‐up; among the 458 remaining patients, median follow‐up was 17 months (range 1–103), the longest follow‐up corresponding to CLL patients (24 months, range 1–98) and the shortest to Diffuse Large B‐cell Lymphoma (DLBCL) (5 months, range 0–25).

### 3.1 Characteristics of the cohort

Patient baseline characteristics are shown in Table 1. The most frequent underlying cancer was CLL in 219 patients (48%). Only 64 patients (12%) had previously undergone autologous hematopoietic stem cell transplantation (auto‐HSCT) and 10 (2%) allogeneic hematopoietic stem cell transplantation (allo‐HSCT). Up to 16% of cases had either neutropenia (severe in 34.7% of them) or lymphopenia, which were receiving corticosteroid treatment at the beginning of the targeted therapy; and immunoglobulin deficiency was present in 26% of cases.

**TABLE 1:** Characteristics of the cohort

Characteristics	*N* = 458		
Age, median (range)	64 (16–91)		
Women; *n* (%)	167 (36%)		
Prior treatments, median (range)	2 (0–9)		
Malignancy type	*N*	%	
Chronic lymphocytic leukemia	219	48	
Mantle cell lymphoma	54	12	
Diffuse large B‐cell lymphoma	33	7	
Follicular lymphoma	34	7	
Waldenström's macroglobulinemia	38	8	
Hodgkin lymphoma	59	13	
T lymphoma	21	4.5	
Medical comorbidities	*N*	%	
Hypertension	29	6	
Diabetes	49	11	
Chronic kidney disease	19	4.5	
Pulmonary disease	21	4	
Autoimmune disease	15	3	
Liver disease	14	3	
Solid organ transplant	2	0.5	
Previous malignancy	42	9	
Antimicrobial prophylaxis	*N*	%	
Viral	250	54	
Acyclovir	202	44	
Valacyclovir	48	10	
None	130	28	
*Pneumocystis jirovecii* pneumonia prophylaxis	267	58	
Cotrimoxazole	243	53	
Pentamidine	24	5	
None	222	48	
Antibacterial prophylaxys	*N*	%	
Quinolones	9	2	
None	449	98	
Previous hematological treatment	*N*		%
Rituximab	244		53
Bendamustine	109		24
Fludarabine based	66		14
CHOP	121		26
Alemtuzumab	3		0.6
Corticosteroids	76		16
Previous stem cell transplantation	71		15
Target drug	Monotherapy *N* (%)		Combination *N* (%)
Ibrutinib	263 (58)		27 (6)
Brentuximab	40 (9)		37 (8)
Idelalisib	26 (6)		11 (2.5)
Obinutuzumab	0		31 (6.7)
Nivolumab	8 (2)		7 (1.5)
Ofatumumab	0		3 (1)
Acalabrutinib	2 (0.5)		0
Venetoclax	1 (0.2)		1 (0.2)
Pembrolizumab	1 (0.2)		0

CHOP, Cyclophosphamide, Doxorubicin, Vincristine, Prednisone.

### 3.2 Targeted therapy and prophylaxis

Median exposure to targeted drug was 8 months (range 1–72). The most frequently administered drug in monotherapy was ibrutinib in 263 (58%) patients, brentuximab in 40 (9%), and idelalisib in 26 (6%) patients, with the most frequent drug administered in combination being brentuximab (37 patients, 8%), mostly with bendamustine. The median number of prior treatment regimens was 2 (range, 1–9), while 42% of patients underwent 3 or more lines of treatment before targeted therapy. The most common prior therapy was rituximab in 244 patients (53%) (see Table 1 for other previous therapy lines and details of targeted drugs treatment).

PJP prophylaxis was administered to 267 patients (58%), due to different reasons (heavily pretreated patients, current steroid treatment, and/or aggressive lymphoma). Antibacterial prophylaxis was almost absent (only 9 patients were treated with levofloxacin). Two‐hundred and two patients received acyclovir (44%) and 48 (10%) valacyclovir as antiviral prophylaxis. HBV prophylaxis was used in 2 patients (1 tenofovir, 1 lamivudine). Two patients were under treatment with isoniazid for latent TBC and one patient received isavuconazole as secondary antifungal prophylaxis for a previous IFI.

### 3.3 Characteristics of episodes of severe infection

#### 3.3.1 Incidence

One‐hundred forty‐seven severe infections developed in 105 patients (23%) during follow‐up. Twenty‐six infectious episodes developed in 20/115 patients treated with targeted drugs in first line (23%).

**FIGURE 1:**
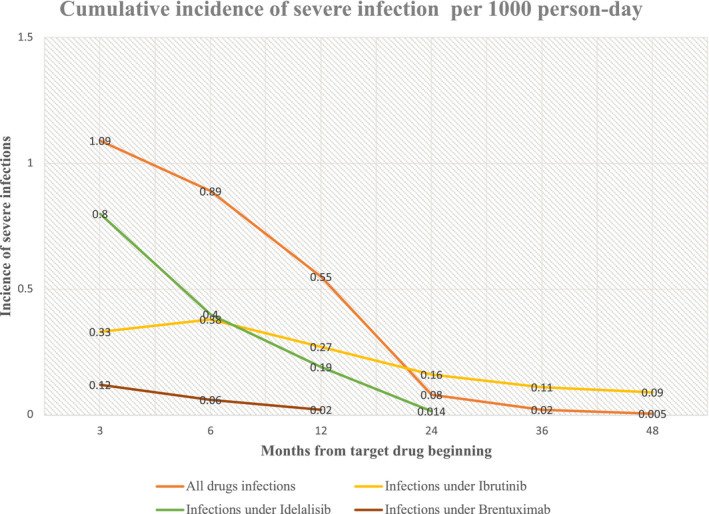
Cumulative incidence of severe infections

The infection incidence was higher in the first 3–6 months of targeted drug treatment (1.09 and 0.89, respectively, per 1000 person‐day) and it decreased from 0.55 infections per 1000 person‐day during the first year of targeted therapy to 0.08 infections per 1000 person‐day during the second and the incidence continued to decrease over time (Figure 1). Focusing on the type of targeted drug, ibrutinib presented the highest CI of severe infections during the first year of treatment (0.27 per 1000 person‐day), followed by idelalisib (0.19 per 1000 person‐day) and brentuximab (0.02 per 1000 person‐day). All types of drugs maintained a decreasing trend of infection over time, with a higher incidence during the first 6 months of treatment. Of 290 patients under ibrutinib treatment, 71 developed severe infection (24%); 17 out of 77 (22%) taking brentuximab (76% in combination); 9 out of 37 (33%) receiving idelalisib (55% in combination); 7 out of 31 (22%) taking obinutuzumab combinations and 1/1 under venetoclax.

#### 3.3.2 Type of infection

Among 147 infectious episodes of our series, the most frequent were bacterial infections (79 episodes, 54%) and the most commonly identified bacterial pathogens were *E*. *coli* and *S*. *aureus*.

Among the 38 episodes caused by identified Gram‐negative bacteria, only 3/29 (10%) were extended‐spectrum beta‐lactamase producers (ESBL). This information was not available for the remaining 9 episodes. Documented infection sources were mainly pulmonary and urinary; 6 bloodstream and 5 catheter‐related infections were detected. We observed one case of atypical mycobacterial infection. Ibrutinib and brentuximab were the most involved targeted drugs and CLL was the most frequent malignancy. Infections resolved in 77% of cases (Table 2).

**TABLE 2:** Severe bacterial infections characteristics

Bacterial severe infections (79)	*N* (%)
Clinical presentation	
Fever	34 (43)
Respiratory symptoms	35 (44)
Abdominal symptoms	9 (11)
Urinary symptoms	12 (15)
Neurological symptoms	3 (3)
Celulitis	4 (5)
Sepsis	9 (11)
Organ involvement	
Pulmonary	36 (45)
Abdominal/urinary	23 (29)
Bloodstream	6 (7.5)
Skin	4 (5)
Catheter related	5 (6)
Septic embolism	2 (2.5)
CNS	3 (3)
Type of bacteria	
*Staphylococcus aureus*	16 (20)
*Escherichia coli*	16 (20)
Coninfections	6 (7.5)
*Klebsiella pneumoniae*	9 (11)
*Pseudomonas aeruginosa*	8 (10)
*Clostridioides difficile*	3 (3)
*Enterococcus* spp	5 (6)
*Haemophilus influenzae*	3 (3)
*Listeria moncytogenes*	2 (2.5)
*Streptococcus* spp	3 (3)
*Staphylococcus lugdensis*	2 (2.5)
*Coxiella burnetti*	1 (1)
*Proteus mirabilis*	1 (1)
NTM Mycobacteria	1 (1)
*Bacilus cereus*	1 (1)
Target drug	
Ibrutinib	51 (64)
Brentuximab	13 (16)
Idelalisib	8 (10)
Obinutuzumab	5 (6)
Venetoclax	1 (1)
Acalabrutinib	1 (1)
Underlying malignancies	
Chronic lymphocytic leukemia	35 (44)
Mantle cell lymphoma	12 (15)
Follicular lymphoma	8 (10)
Hodgkin lymphoma	7 (8)
Diffuse large B‐cell lymphoma	9 (11)
T lymphoma	4 (5)
Waldenström's macroglobulinemia	4 (5)
Outcome	
Resolved	61 (77)
Dead due to infection	18 (23)

Viral infections represented 19% of total episodes (28/147). Respiratory viruses (including 11 SARS‐CoV‐2 pneumonia, diagnosed during the 17 months of follow‐up of our series) produced most of the episodes. One case of hepatitis B, 4 CMV, 3 HSV, and 1 EBV reactivations were identified and no primary infection was observed. Again, the most commonly involved targeted drugs were ibrutinib (57%) and brentuximab (25%).

Regarding fungal infections, 9 (6%) episodes were recorded, including 8 proved or probable invasive aspergillosis (IA) and 1 *C*. *albicans* bloodstream infection (Table 3). All but one IFI were identified in indolent LPD patients receiving ibrutinib treatment, while the remaining one was reported in a CLL patient under idelalisib; incidence of IFI in ibrutinib patients was 3.3% with a median exposure to targeted drug of 6.2 months (range 2.7–33.1). Median number of prior lines of treatment in this cohort was 2 (range 1–5). *Aspergillus* spp. was the fungus most frequently isolated. Of note, there were no cases of PJP pneumonia in our series.

**TABLE 3:** Characteristics of patients with IFI

Case	Age, sex malignancy	Drug	Days of exposure to the drug prior to infection	No. previous lines	Use of CE	Location	Method of diagnosis	IFI criteria	Drug suspension	Alive
1	63, M,CLL	Ibrutinib	276	2	N	Lung	NA	Probable	Temporary	Y
2	65, F,CLL	Ibrutinib	994	3	N	Lung	*A*. *fumigatus* serum GM	Proven	Indefinite	Y
3	58,M, MCL	Ibrutinib	88	1	Y	Lung+eye	NA	Probable	Indefinite	Y
4	75,M,CLL	Ibrutinib	617	2	N	Lung	calcofluor	Proven	Temporary	Y
5	72,M,WM	Ibrutinib	655	3	N	Disseminated	*C*. *albicans* BAL GM	Proven	Temporary	Y
6	73,M,CLL	Ibrutinib	40	5	Y	Lung	NA	Probable	Indefinite	N
7	77, F,CLL	Idelalisib	188	2	N	Abdominal+lung	*A*. *fumigatus* +*A*. *Terreus* serum GM	Proven	Indefinite	N
8	53,M,CLL	Ibrutinib	81	2	N	Lung	*A*. *fumigatus* serum GM	Proven	Indefinite	Y
9	65,M,WM	Ibrutinib	187	2	N	Brain	*A*. *flavus* brain biopsy	Proven	Indefinite	N

CE, corticosteroids; BAL, bronchoalveolar lavage; GM, galactomannan antigen test.

We described 32 (22%) clinically diagnosed infections without microbiological identification. Among them, the most common presentations were pneumonia and febrile neutropenia. There were 6 polymicrobial severe infections in our series: 5 abdominal sepsis or severe infections caused by *Enterococcus faecium* or *C*. *difficile* in association with other pathogens, 1 patient with coexistence of CMV and *Herpes simplex* reactivation; in all these cases, patients had received 3 or more lines of previous treatment, including bendamustine; moreover, 3 of the 4 patients were treated with corticosteroids (>=1mg/kg or more).

#### 3.3.3 Management

In 114/147 (77%) infections, targeted treatment was temporarily discontinued, while only in 33/147 (22%) discontinuation was permanent. Specifically, in patients with IFI (8), the targeted drug was discontinued temporarily or indefinitely in almost all cases to avoid possible interactions between targeted therapy and antifungal therapy. In patients with SARS‐CoV‐19 pneumonia under ibrutinib treatment (8/11), BTK inhibitor was maintained in 6 patients.

#### 3.3.4 Outcome

Only 10 patients (9.5%) developed 3 or more new episodes of severe infections. Twenty patients (19.5%) developed more than one severe infections, of which 2 where reinfections (*S*. *aureus* and *P*. *aeruginosa* lung infections) in two patients heavily pretreated (2 prior lines of therapy and 2 auto‐HSCT).

A total of 29 patients (6%) died due to the infection; these were mostly cases of bacterial infections (62%) with *S*. *aureus*, *E*. *coli* and *P*. *aeruginosa* as the most frequent pathogens. Median time from the beginning of the new drug to death was 7 months (range 0–54); lymphoid cancer was in progression in 19 patients (58%) who died. Two deaths occurred among patients with IFI, only one was attributable to IFI (Table 3).

One out of the 8 patients who developed SARS‐CoV19 pneumonia undergoing ibrutinib died due to the infection: ibrutinib was not suspended during the infection episode.

### 3.4 Risk of severe infection

When comparing patients with and without severe infection, severe lymphopenia (*p* = 0.009, OR 4.7, CI 1.3–1.7), combined targeted treatment vs. monotherapy (*p* = 0.014, OR 2.2, CI 1.1–4.2) and previous rituximab (*p* = 0.03, OR 1.8, CI 1.05–3.3) were more frequently associated with infection. In multivariate analysis, all these parameters remained significant. Type of LPD, presence of CLL, steroids, previous number of treatment lines among others, were not significant infection risk factors, either in univariate or multivariate analysis in our study (Table 4).

**TABLE 4:** Infection risk analysis: patients with severe infections versus those with no infection

Parameter	Univariate analysis	*p* value OR (95% CI)	Multivariate analysis	*p* value OR (95% CI)
Age	0.4	1.2 (0.6–2.3)		
Female sex	0.18	1.6 (0.8–2.6)		
CLL as underlying cancer	0.7	0.9 (0.5–1.6)		
Ibrutinib as target treatment	0.39	1.2 (0.7–2.3)		
3 or > prior treatment regimen	0.71	0.9 (0.5–1.5)		
Prior fludarabine	0.44	1.3 (0.6–2.8)		
Prior bendamustine	0.97	0.9 (0.5–1.8)		
Prior rituximab	0.03	1.8 (1.05–3.3)	0.036	2.3 (1.05–5.1)
Prior alemtuzumab	0.35	0.5 (0.4–0.6)		
Neutropenia <1500	0.7	1.1 (0.5–2.3)		
Neutropenia <500	0.3	1.7 (0.5–6.0)		
Lymphopenia <800	0.009	4.7 (1.3–17)	0.016	4.7 (1.3–17)
Corticosteroids use	0.5	1.19 (0.6–2.3)		
PJP prophylaxis	0.5	1.2 (0.6–2.2)		
Hepatis disease	0.2	1.2 (0.5–3.0)		
Diabetes	0.28	1.5 (0.6–3.8)		
Previous cancer	0.61	1.2 (0.5–3.0)		
Aggressive vs indolent LPD	0.68	0.8 (0.4–1.5)		
Previous SCT	0.64	0.4 (0.2–1.05)		
Combined vs single treatment	0.014	2.2 (1.1–4.2)	0.006	3.1 (1.3–7.1)

CLL, chronic lymphocytic leukemia; PJP, Pneumocystis jirovecii pneumonia; LPD, lymphoproliferative diseases; SCT, stem‐cell transplantation.

Focusing on infectious etiology, previous use of rituximab (*p* = 0.013, OR 2.1, CI 1.1–3.9) and severe lymphopenia (*p* = 0.004, OR 4.5 CI 1.2–1.8) were significantly associated with the development of bacterial infection both in univariate and multivariate analysis. Again, rituximab (*p* = 0.007, OR 0.3 CI 0.1–0.7) and fludarabine (*p* = 0.024, OR 2.6 CI 1.1–6.2) prior use seemed to be a risk factor for viral infection. Ibrutinib treatment was associated with viral infection in the univariate analysis but did not remain significant in the multivariate.

Previous use of alemtuzumab (*p* < 0.001, OR 0.36, CI 0.018–0.075) was the only risk factor for development of IFI in our series in univariate analysis but was not significant in multivariable. In 10 patients of the series who presented 3 or more severe infections, the only significant risk factor was the presence of 1 or more comorbidities (*p* = 0.03, OR 1.6, CI 0.4–4.5), being the most frequent the chronic respiratory diseases.

## 4 DISCUSSION

This is one of the largest series to describe infection incidence in a general population of patients with lymphoproliferative diseases receiving all types of targeted therapy in a real‐life setting. We found a cumulative incidence of 0.55 infections per 1000 person‐days during the first year of targeted therapy, with higher rates in the first 3–6 months of therapy, accounting for a 23% incidence of severe infections during a median follow‐up of 17 months among patients with lymphoid cancer who received targeted drugs. We identified risk factors for the development of severe infections that include previous use of rituximab, combined treatment and severe lymphopenia. Moreover, pretreated patients seemed to be at higher risk for bacterial and viral infections.

During the chemotherapy era, an incidence of bacterial infections ranging from 18% to 36% was described with fludarabine or bendamustine combination.^31^, ^32^, ^33^, ^34^ Our findings confirm that the incidence of severe infections with the new targeted therapies is not superior to that of prior chemotherapeutic regimens. Interestingly, severe infections were more common during the first six months of treatment, but then decreased progressively. We consider this to reflect the strong influence in the incidence of infection of previous treatments and uncontrolled hematological malignancy status at the beginning of the targeted drug treatment. As the underlying disease becomes controlled under the new treatment, infection progressively subsides. The majority of infections were seen in ibrutinib patients, therefore the contribution of the ITK in promoting immune reconstitution after the first 6 months of therapy must be taken into consideration: increasing levels of IgA have been described after the first year of ibrutinib treatment,^35^ as well as ITK inhibition^36^ that leads to recovery of TCR repertoire diversity^37^ by T‐cell reset with improvement of immunologic synapsis, reduction of Th2 and PD1 expression and conversely, Th1 increase.

Our results support those reported by Varughese^7^ who describes 11.4% of infections occurring during the first year of ibrutinib treatment in lymphoid cancer patients, with a predominance of bacterial infections, *S*. *aureus* being the most frequent pathogen, respiratory tract infection the most frequent clinical presentation and presenting a similar incidence of IFI as our series. The majority of patients in our series received ibrutinib and our findings coincide with those of Varughese.^7^ In contrast, we showed a higher incidence of severe infections (26% vs. 11.4%).

In patients treated with idelalisib, clinical trials^28^, ^38^ show a 5‐fold increase in PJP infection risk. All patients under idelalisib in our series received PJP prophylaxis and no PJP pneumonia was reported. CMV reactivation was observed in 3.8% of these patients, similar to that described in clinical trials.

According to clinical trials, brentuximab vedotin does not seem to increase infectious risk,^39^, ^40^ even though neutropenia is a common adverse effect; in contrast, in our series, we found a non‐negligible cumulative incidence of infection, with severe bacterial infections under brentuximab. This could be due to a heavily pretreated population and uncontrolled hemopathy, as 76% of infections happened in the first 2 months of treatment.

No severe infections developed in patients under immune checkpoint inhibitors, suggesting that those targeted drugs are not directly related to higher risk of infection. Patients under treatment with acalabrutinib, ofatumumab, venetoclax, pembrolizumab, or nivolumab are scarce in our series, therefore more data are needed to draw a conclusion about infection risk of those drugs.

There is a concern specifically regarding increased incidence of IFI in patients treated with targeted therapies. In the present series, incidence of IFI was lower than described in the literature (0.5%–18%).^3^, ^7^, ^41^, ^42^, ^43^, ^44^ In particular, we did not record any case of PJP pneumonia in contrast with other reports.^9^, ^11^, ^12^ Of note, almost all IFI cases in our series were diagnosed in CLL patients under ibrutinib with similar incidence to what has been reported before (2.5%–4%),^44^, ^45^ and presence of other known risk factors such as receipt of >3 prior treatment regimens and/or corticosteroids (Table 3). However, no specific independent risk factors for IFI were found in the statistical analysis.

Infection conveyed the need for discontinuation of the novel drug in a high proportion of cases, which can impact outcome in the long term. Only 22% of cases required a definitive discontinuation, however in most of the cases (69%) discontinuation was temporary. The mortality rate among patients with severe infection was 6%. The role of infection in mortality is difficult to ascertain. Most of the deaths occurred soon after the introduction of the targeted therapy (median of 7 months), which points to the role of uncontrolled hematological disease as a co‐trigger of mortality.

There is a need for prevention of infection to mitigate the consequences of infection, yet there remains a lack of information regarding specific risk factors for infection in LPD patients treated with targeted therapy. The study by Varughese^7^ described the association of neutropenia and 3 or more previous treatment lines as significant risk factors for severe infection in patients treated with ibrutinib, and use of corticoids and >3 lines of previous treatment were related specifically to IFI infections; moreover, comorbidities as diabetes or liver disease have been described as risk factors for opportunistic infections (OI) in ibrutinib patients.^29^ In view of this lack of information, there are few definite recommendations for antimicrobial prophylaxis in patients with LPD treated with targeted therapies.^46^ Besides hepatitis B prophylaxis during treatment with anti‐CD20, a recent position paper^47^ proposes PJP prophylaxis during BTK or PI3K inhibitors and immune checkpoints inhibitors when glucocorticoids medications exceeds 3–4 weeks. CMV monitoring is suggested with anti‐CD30 antibodies and idelalisib. No specific prophylaxis is recommended with ibrutinib treatment, in spite of a trend to an increased incidence of fungal infections in these patients.

Our opinion is that heavily pretreated patients, and/or those with severe lymphopenia and/or targeted drug used in combination, should be closely monitored and considered for viral prophylaxis with acyclovir, at least during the first 6 months of treatment with idelalisib or ibrutinib. Antibacterial prophylaxis is controversial, considering the concern for development of antibiotic resistance, and should be evaluated on a case‐by‐case basis in high‐risk patients, where benefits outweigh the risks. Regarding IFI, as incidence in the present series is below the 10% threshold, and furthermore, the current drugs used as prophylaxis interact with ibrutinib through inhibition of CYP3A4, we consider that antifungal prophylaxis is not indicated. In CLL patients treated with ibrutinib, with >2 prior lines, or idelalisib, we suggest careful monitoring and considering periodical screening (GM, PCR etc.) during the first 6 months of treatment. For PJP, an important proportion of cases received prophylaxis according to the protocol of each hospital and no PJP was reported in our series, confirming the effectiveness of prophylaxis. Accordingly, we agree on PJP prophylaxis indication as per the international peer‐reviewed guidelines.^26^, ^47^

Limitations of this study include retrospective design, dependence on the accuracy of electronic medical records and possible selection bias. Moreover, heterogeneity of underlying disease and drug exposure could be another limitation, as a confounding factor for infection risk in the series. On the other hand, our contribution is the first multicentric and cooperative series of LPD treated with different target drugs in real life, giving a real‐world point of view of current clinical practice, with a follow‐up of 17 months. We consider the results generalizable because they come from different clinical scenarios.

In conclusion, a high proportion of patients in our series presented severe infections during follow‐up, with non‐negligible attributable mortality, but infection incidence is not superior to that observed during the chemotherapy era. In selected cases with specific risk factors for infection, close monitoring and antimicrobial prophylaxis should be considered.

## 5 CONFLICT OF INTEREST

The authors have no conflicts of interest to disclose.
